# Effects of a swallowing and oral care intervention for patients following endotracheal extubation: a pre- and post-intervention study

**DOI:** 10.1186/s13054-019-2623-2

**Published:** 2019-11-09

**Authors:** Chung-Pei Wu, Yu-Juan Xu, Tyng-Guey Wang, Shih-Chi Ku, Ding-Cheng Chan, Jang-Jaer Lee, Yu-Chung Wei, Tzu-Yu Hsiao, Cheryl Chia-Hui Chen

**Affiliations:** 1 Department of Nursing, National Taiwan University Hospital and National Taiwan University College of Medicine, 1, Jen-Ai Rd., Section 1, Taipei, Taiwan, Republic of China 100; 2 Department of Physical Medicine and Rehabilitation, National Taiwan University Hospital and National Taiwan University College of Medicine, Taipei, Taiwan, Republic of China; 3 Department of Internal Medicine, National Taiwan University Hospital and National Taiwan University College of Medicine, 1, Jen-Ai Rd., Section 1, Taipei, Taiwan, Republic of China 100; 4 Department of Geriatrics and Gerontology, National Taiwan University Hospital and National Taiwan University College of Medicine, Taipei, Taiwan, Republic of China; 5 Superintendent’s Office, National Taiwan University Hospital Zhu-Dong Branch, Hsinchu, Taiwan, Republic of China; 6 Department of Dentistry, National Taiwan University Hospital and National Taiwan University College of Medicine, Taipei, Taiwan, Republic of China; 7 Department of Statistics, Feng Chia University, Taichung, Taiwan, Republic of China; 8 Department of Otolaryngology, National Taiwan University Hospital and National Taiwan University College of Medicine, Taipei, Taiwan, Republic of China

**Keywords:** Endotracheal intubation, Dysphagia, Critical illness, Oral intake, Swallowing, Salivation

## Abstract

**Background:**

For patients who survive a critical illness and have their oral endotracheal tube removed, dysphagia is highly prevalent, and without intervention, it may persist far beyond hospital discharge. This pre- and post-intervention study with historical controls tested the effects of a swallowing and oral care (SOC) intervention on patients’ time to resume oral intake and salivary flow following endotracheal extubation.

**Methods:**

The sample comprised intensive care unit patients (≥ 50 years) successfully extubated after ≥ 48 h endotracheal intubation. Participants who received usual care (controls, *n* = 117) were recruited before 2015, and those who received usual care plus the intervention (*n* = 54) were enrolled after 2015. After extubation, all participants were assessed by a blinded nurse for daily intake status (21 days) and whole-mouth unstimulated salivary flow (2, 7, 14 days). The intervention group received the nurse-administered SOC intervention, comprising toothbrushing/salivary gland massage, oral motor exercise, and safe-swallowing education daily for 14 days or until hospital discharge.

**Results:**

The intervention group received 8.3 ± 4.2 days of SOC intervention, taking 15.4 min daily with no reported adverse event (coughing, wet voice, or decreased oxygen saturation) during and immediately after intervention. Participants who received the intervention were significantly more likely than controls to resume total oral intake after extubation (aHR 1.77, 95% CI 1.08–2.91). Stratified by age group, older participants (≥ 65 years) in the SOC group were 2.47-fold more likely than their younger counterparts to resume total oral intake (aHR 2.47, 95% CI 1.31–4.67). The SOC group also had significantly higher salivary flows 14 days following extubation (β = 0.67, 95% CI 0.29–1.06).

**Conclusions:**

The nurse-administered SOC is safe and effective, with greater odds of patients’ resuming total oral intake and increased salivary flows 14 days following endotracheal extubation. Age matters with SOC; it more effectively helped participants ≥ 65 years old resume total oral intake postextubation.

**Trial registration:**

NCT02334774, registered on January 08, 2015

## Introduction

For patients who survive a critical illness and have their oral endotracheal tube removed, dysphagia is highly prevalent and may persist far beyond hospital discharge [1]. Dysphagia after extubation affects up to 62% of intensive care unit (ICU) patients, especially for patients with prolonged (≥ 48 h) endotracheal intubation [2–6]. When dysphagia persists, resumption of oral intake is delayed, committing patients to feeding tube dependence [6, 7]. In one large observational trial (*N* = 933) from Switzerland, dysphagia lasted until hospital discharge in 60.4% of adult ICU patients [2]. By 21 days after extubation, even for ICU patients without prior swallowing difficulties or known pathologies such as stroke or neuromuscular deficits, 15.5% had persistent dysphagia, could not resume oral intake, and were feeding tube dependent [6]. Indeed, without intervention, dysphagia symptoms were sustained far beyond hospital discharge; 23% of orally intubated ICU survivors had dysphagia persisting > 6 months in a 5-year longitudinal cohort study involving 13 ICUs at 4 US teaching hospitals [8].

Dysphagia after extubation negatively affects patient outcomes [1], leading to delayed resumption of oral intake [6, 7, 9], poor life quality [10, 11], aspiration pneumonia [4, 8, 10, 12], longer ICU and hospital stays [2, 7], and increased 90-day mortality [2]. Evidence for dysphagia treatment, however, is limited [2] as few intervention studies have been designed to reduce dysphagia or the time needed to resume total oral intake after extubation [13].

Herein, we hypothesized that improving oral lubrication, oral sensation, and strength in the lips, tongue, jaw, and cheeks would reduce time to resume total oral intake and enhance salivary flow for patients who received prolonged endotracheal intubation and had no prior swallowing difficulties or known pathologies such as stroke or neuromuscular deficits.

Our previous studies of the sequelae of prolonged endotracheal intubation in this group of ICU patients [6, 14, 15] revealed that reduced salivary flow [14], sensorimotor impairment of the tongue [15], poor lip seal [14], and restricted mouth opening (i.e., weakness of masticatory muscles to move the jaw) [14] were highly prevalent and may persist 14 days postextubation. As these sequelae contribute to dysphagia and delay oral intake after extubation [1, 16], we developed a nurse-administered, hospital-based (provided daily until hospital discharge, up to 14 days) swallowing and oral care (SOC) intervention comprising toothbrushing/salivary gland massage, oral motor exercise (i.e., range of motion [ROM] exercises for the lips, tongue, jaw, and cheeks), and safe-swallowing education.

We hypothesized that toothbrushing/salivary gland massage would enhance oral lubrication and oral sensation by mechanically stimulating oral sensory receptors and increasing salivary flow [17, 18]. Oral motor exercise may alleviate patients’ poor lip seal, reduced tongue strength, poor lingual agility, and restricted mouth opening by strengthening the lips, tongue, jaw, and cheeks [19, 20]. While these two SOC protocols may facilitate recovery from the sequelae of prolonged endotracheal intubation, safe-swallowing education is important to include as a safety precaution to reduce the aspiration risk [16].

As primary endpoints, time to resume total oral intake (i.e., total oral diet with multiple consistencies, measured by ≥ level 6 on the functional oral intake scale [FOIS] and censored 21 days postextubation) and unstimulated whole-mouth salivary flow (measured by oral Schirmer’s test 2, 7, and 14 days postextubation) were measured by blinded nurses. Swallowing was not evaluated instrumentally (i.e., videofluoroscopy or fiberoptic endoscopic evaluation), since these tests are invasive and our study was a pilot. Intervention feasibility was also evaluated based on patients’ adherence to the SOC intervention, time spent providing SOC, and adverse events during the intervention.

## Methods

### Study design, settings, and participants

**Fig. 1:**
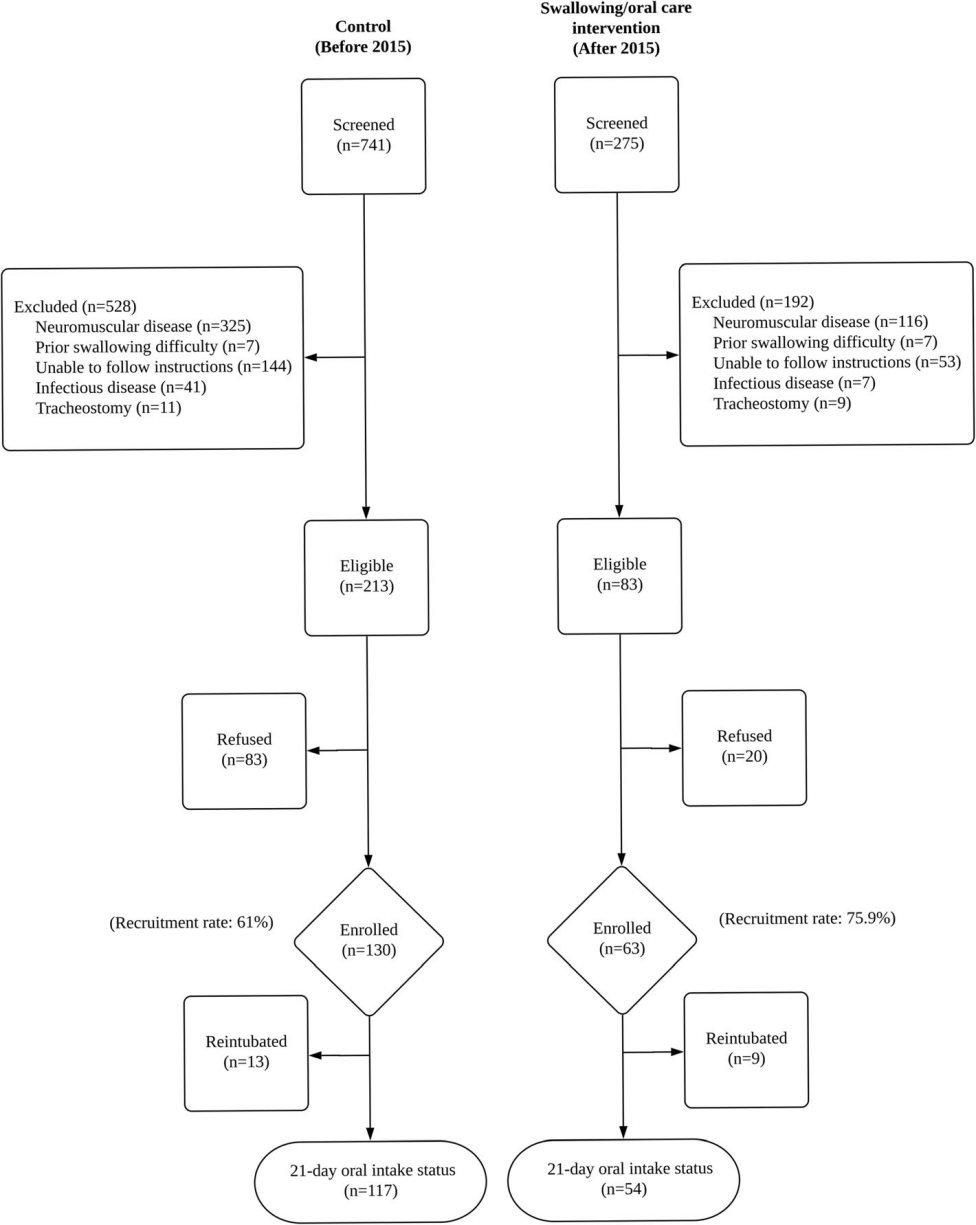
Study flow diagram

This pre- and post-intervention study with historical controls was conducted at a tertiary medical center in Taipei, Taiwan. From October 2012 to November 2015, participants were recruited from consecutive patients (≥ 50 years) admitted to the medical center’s six medical ICUs if they had received emergency oral endotracheal intubation for at least 48 h. Patients were excluded if they (1) had a history of neuromuscular disease (e.g., stroke, Parkinsonism) or head and neck deformities, (2) had a preexisting swallowing difficulty, (3) received tracheostomy, (4) could not respond to questions/intervention, and (5) were absolutely quarantined (e.g., for open or infectious tuberculosis). All patients and/or their legal representative signed written informed consent. Participants recruited before 2015 served as controls; participants recruited after 2015 received usual care plus the SOC intervention and served as the intervention group (Fig. 1). As no prior studies on this topic were available, we could not estimate the intervention effect to determine the study size. Nonetheless, we included 54 participants in the SOC group. The study was approved by the Human Research Ethics Committee of the study hospital (201411079RIND) and registered at the Clinical Trials Registry (trial no: NCT02334774).

### Usual care

Usual care consisted of standard hospital care provided by ICU physicians and nurses. Upon extubation, oral intake was withheld until participants demonstrated no signs of choking (i.e., coughing, drooling, wet voice, or decreased oxygen saturation) on a small amount of water, progressing to small and consistent amounts of food/liquid as tolerated. All participants were encouraged to take food/liquid orally and did so as tolerated. A speech therapist or dietician provided additional care only at the attending physician’s request. Oral care was provided each shift by ICU nurses using oral swabs and rinsing with 2% chlorhexidine gluconate. Once patients were transferred to a general ward, oral care was considered a self-care process that was often assisted by family members.

### SOC intervention

After intervention group participants were successfully extubated, the SOC intervention was administered on the next day (regardless of intake status) and daily until hospital discharge or 14 days postextubation (whichever occurred first). This intervention schedule was based on our observation that sequelae of endotracheal intubation may persist 14 days [14]. A trained SOC nurse, equipped with cheek retractor, dental suction tube, and tongue holder, provided the SOC intervention, i.e., toothbrushing/salivary gland massage, oral motor exercise, and safe-swallowing education (Additional file 1)*.* Briefly, the SOC nurse brushed participants’ oral cavity (teeth/gum, tongue, and palate) with a soft toothbrush using distilled water to remove the coated plaque, mechanically stimulate tissues, and rinse the oral cavity. The SOC nurse then moisturized participants’ lips with Vaseline® before placing fingers on participants’ cheeks and gently massaging/pressing the surface overlying the parotid, sublingual, and submandibular salivary glands. Participants were then asked to purse the lips, move the tongue, open the mouth widely, and inflate the cheeks (each with 3, 5, or 10 repetitions/with or without resistance, depending on participants’ tolerance) as one round of ROM exercises for the lips, tongue, jaw, and cheeks. Based on participants’ intake status, brief safe-swallowing education was offered daily, explaining the signs and symptoms of unsafe swallowing and providing tips on sitting up to eat and modifying dietary texture and viscosity for patients and their family caregivers to reduce the aspiration risk. Before implementing the intervention, the SOC nurse was trained on-site for 2 months by experienced ICU nurses, a physician in rehabilitative medicine, and speech-language pathologists. Speech-language pathologists coached SOC nurse on identifying adverse events (such as wet voice) and gave practical tips on salivary gland massage and oral motor exercises of the lips, tongue, jaw, and cheeks in the ICU setting.

### Data collection and outcome measures

Data on participant characteristics (age, gender, education [years], current smoker [yes/no], body mass index, admission diagnosis [respiratory failure, septic shock/sepsis, heart disease, gastrointestinal bleeding, other], illness severity [Acute Physiological and Chronic Health Evaluation II (APACHE II) 0–24, ≥ 25]) were abstracted from medical records. Baseline data on oxygen supplementation (simple mask, non-rebreathing mask, bi-level positive airway pressure [yes/no]), intake level (FOIS level 1, levels 2–3, levels 4–7 to indicate nothing by mouth, tube feeding with intake attempts, and oral intake), and salivary flow (cm/5 min) were evaluated by a research nurse at enrollment (before intervention).

All participants’ daily intake status was evaluated for 21 days postextubation by a research nurse using the FOIS, a validated tool with established validity (81–98%) and inter-rater reliabilities (0.86 to 0.91) [21]. FOIS scores range from levels 1 to 7, with levels 1 through 3 indicating varying degrees of tube feeding and levels 4 through 7 indicating varying degrees of oral intake [21]. We considered FOIS level 6 (total oral diet with multiple consistencies, without special preparation but with specific food limitations) or 7 (total oral diet with no restriction) as “resume total oral intake.” Unstimulated whole-mouth salivary flow was evaluated 2, 7, and 14 days postextubation using the oral Schirmer’s test [22]. Considering response burden and time required for changes, the oral Schirmer’s test, unlike the FOIS, was not measured daily. With participants sitting upright, research nurses held a standardized 1-cm-wide by 17-cm-long Schirmer’s tear test strip vertically, with the rounded end of the strip at the floor of their mouth. At the end of 5 min, a wetting length in centimeter was recorded.

Feasibility of the SOC intervention was evaluated by time spent providing the SOC, patients’ adherence to SOC components, and adverse events (i.e., coughing, wet voice, and decreased oxygen saturation) during and immediately after the intervention. Adherence was calculated as the percentage of the session that a participant completed compared to the maximal session that a participant could receive (maximum 14). Rates were documented separately for three SOC components (toothbrushing/salivary gland massage, oral motor exercise, and safe-swallowing education).

### Statistical analysis

Participants’ characteristics were analyzed by frequency (percentage) and mean (standard deviation) or median (interquartile range). Differences in characteristics by group were analyzed using the non-parametric Mann-Whitney *U* test for continuous variables and Fisher’s exact test for categorical variables.

Time to resume total oral intake for the SOC and control groups was plotted using the Kaplan-Meier survival curves. Considering age and baseline-cohort differences, participants’ between-group clinical characteristics that differed (*P* < 0.1, Table 1) were entered as fixed covariates (non-time dependent) in Cox proportional hazard modeling and reported as adjusted hazard ratios (aHR). Salivary flows 2, 7, and 14 days following extubation were compared between the SOC and control groups by generalized estimating equations (GEE). An interaction term, “time by group,” was used to indicate the between-group slope difference over time. Analyses were performed on a per-protocol approach, and no data were imputed. All analyses were performed using IBM SPSS Statistics (version 21, 2012, IBM Corporation). Significance was set at *P* < 0.05.

**Table 1:** Participant characteristics by group

Characteristic	SOC (*n* = 54)	Control (*n* = 117)	*P* value
Demographic			
Age, years, mean (SD)	71.4 (10.8)	68.2 (10.8)	0.39^a^
Female gender, *n* (%)	21 (38.9)	42 (35.9)	0.74^b^
Education, years, mean (SD)	9.4 (5.3)	9.9 (4.8)	0.94^a^
Current smoker, *n* (%)	9 (17.0)	30 (25.9)	0.24^b^
Body weight, mean (SD)	59.5 (11.5)	64.1 (15.1)	0.44^a^
Body mass index, mean (SD)	22.9 (4.0)	23.8 (5.3)	0.49^a^
Clinical			
Admission diagnosis, *n* (%)			0.83^b^
Respiratory failure	23 (42.6)	53 (45.3)	
Septic shock/sepsis	14 (25.9)	28 (23.9)	
Heart disease	12 (22.2)	18 (15.4)	
Gastrointestinal bleeding	3 (5.6)	8 (6.8)	
Other	2 (3.7)	10 (8.5)	
APACHE II score, *n* (%)			0.13^b^
0–24	26 (48.1)	72 (61.5)	
≥ 25	28 (51.9)	45 (38.5)	
Intake level, *n* (%)			< 0.01^b^
FOIS level 1	34 (63.0)	48 (41.0)	
FOIS levels 2–3	17 (31.5)	33 (28.2)	
FOIS levels 4–7	3 (5.6)	36 (30.8)	
Oxygen supplementation, *n* (%)	38 (70.4)	62 (53.9)	0.05^b^
Salivary flow, cm/5 min, median (IQR)	3.0 (3.0)	3.9 (2.0)	0.03^a^

*FOIS* functional oral intake scale, *SOC* swallowing and oral care, *APACHE* Acute Physiological and Chronic Health Evaluation. FOIS Level 1, nothing by mouth; level 2, tube dependent with minimal attempts of food or liquid; level 3, tube dependent with consistent oral intake of food or liquid; level 4, total oral diet of a single consistency; level 5, total oral diet with multiple consistencies, but requiring special preparation or compensations; level 6, total oral diet with multiple consistencies with special preparation, but with specific food limitations; level 7, total oral diet with no restrictions

^a^Mann-Whitney *U* test

^b^Fisher’s exact test

## Results

The SOC intervention was successfully implemented with differences in baseline characteristics noted between the SOC and control groups (Table 1). Namely, participants in the SOC group were older (71.4 ± 10.8 years) than controls (68.2 ± 10.8 years, *P* = 0.39) and their body weight (59.5 ± 11.5 kg) was lower than that of controls (64.1 ± 15.1 kg, *P* = 0.44). Within 48 h postextubation, a higher percentage of SOC participants received oxygen supplementation (70.4%) than controls (53.9%, *P* = 0.05). Most importantly, baseline intake level was significantly worse for the SOC group (63.0% were at FOIS level 1 [nothing by mouth]) than for the control group (41.0% were at FOIS level 1; *P* < 0.01). Conversely, only 5.6% of the SOC group vs. 30.8% of controls were at FOIS levels 4–7. Notably, no participant’s intake was withheld due to gastrointestinal causes such as bleeding, although 11 participants were diagnosed at admission with gastrointestinal bleeding. As to salivary flow, the SOC group also had a significantly lower baseline salivary flow (3.0 cm/5 min) than the controls (3.9 cm/5 min, *P* = 0.03).

### Resume total oral intake

Overall, 45.6% of participants did not resume total oral intake by 21 days postextubation, but the SOC group took less time to resume total oral intake (14 ± 4.3 days) than controls (16 ± 2.2 days) although this difference did not reach significance. After adjusting for three baseline between-group differences (age, intake level, oxygen supplementation), the SOC group was significantly more likely to resume total oral intake (aHR 1.77, 95% CI 1.08–2.91) than controls (Table 2). Stratified by age group, older participants (≥ 65 years) who received the SOC intervention had a 2.47-fold significantly higher likelihood of resuming total oral intake (aHR 2.47, 95% CI 1.31–4.67) than their younger counterparts (50 to 64 years).

**Table 2:** Time to resume total oral intake by group

Participants	SOC (*n* = 54)	Control (*n* = 117)	Adjusted hazard ratio^b^
	Median time, days^a^	Median time, days^a^	[95% confidence interval]
All participants	14	16	1.77* [1.08, 2.91]
Age ≥ 65 years (*n* = 36/74)	14	21	2.47* [1.31, 4.67]
Age < 65 years (*n* = 18/43)	9	12	1.32 [0.55, 3.16]

^a^Median time to reach FOIS level 6, based on the Kaplan-Meier analysis

^b^Adjusted for age, baseline intake level (FOIS level 1; levels 2–3; levels 4–7), oxygen supplementation via Cox proportional hazard modeling

*P < 0.05

### Salivary flow over 14 days following extubation

**Fig. 2:**
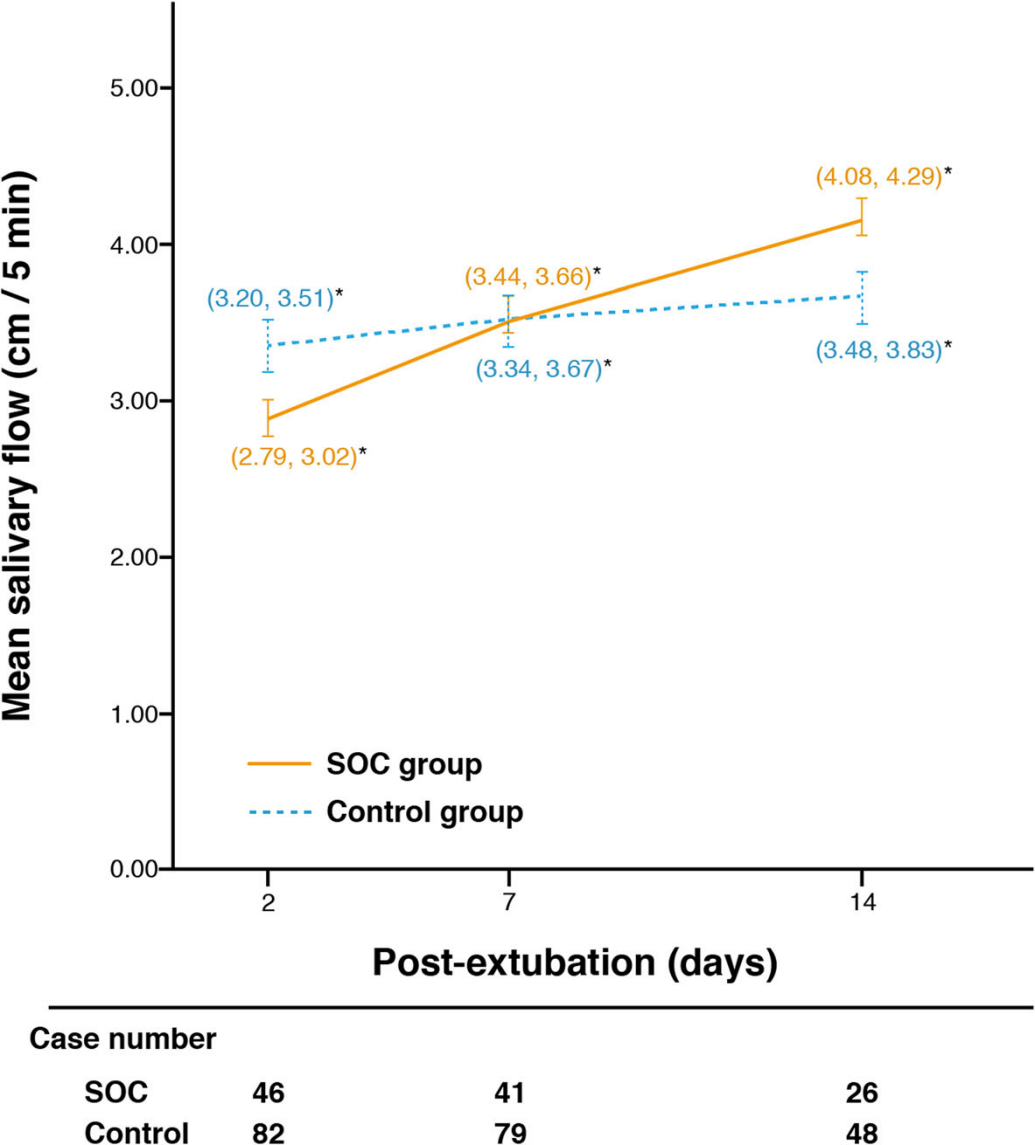
Salivary flow 2, 7, and 14 days after extubation by group. SOC, swallowing and oral care. *95% confidence interval

Before the intervention, 37.4% of participants met the criterion for hyposalivation (salivary flow ≤ 3 cm/5 min) 2 days postextubation. GEE analysis revealed that the SOC group had greater salivary flow over 14 days following extubation (β = 0.67, 95% CI 0.29–1.06, *P* < 0.01) than controls. Figure 2 shows the mean salivary flow for the SOC and control groups 2, 7, and 14 days after extubation.

### Intervention feasibility

Participants and their caregivers positively perceived the SOC intervention, which took on average 15.4 min to complete daily. Participants received on average 8.3 ± 4.2 days of the SOC (range 1–14), with no reported adverse events (coughing, wet voice, or decreased oxygen saturation) during and immediately after the intervention. Adherence to SOC components was moderately good; for the sessions that participants could receive (maximum 14), adherence rates were 95.3% for toothbrushing/salivary gland massage, 70.0% for oral motor exercise, and 80.1% for safe-swallowing education, for an overall adherence rate of 81.8%. Notably, most participants could not exercise oral motor muscles against resistance due to their weakness, dyspnea, non-invasive ventilation, and/or medication issues until 3 to 4 days after extubation, resulting in a lower adherence rate of 70.0%. However, before the intervention ended, approximately 80% could exercise against resistance, and among those, 10% could only do 3 repetitions, 20% could do 5 repetitions, and 70% could do 10 repetitions in the oral-motor exercise protocol.

## Discussion

The most important finding of this study was that our nurse-administered SOC intervention effectively increased patients’ odds of resuming total oral intake and increased salivary flow 14 days following extubation after prolonged (≥ 48 h) endotracheal intubation. This finding is important because patients who were successfully extubated after ≥ 48 h endotracheal intubation often complained of dry mouth and had difficulty resuming total oral intake [14, 23]. Our SOC intervention, offered on average for 15.4 min daily over 8 days, kept patients’ oral cavity moist and clean; their lips, tongue, and jaw were moving freely; and patients were well-informed on safe-swallowing strategies, thus increasing their odds of resuming total oral intake 1.77-fold.

Age mattered, with a stronger intervention effect for patients at least 65 years old; older participants who received the SOC were 2.47-fold more likely to resume total oral intake than their younger counterparts (50 to 64 years). Moreover, salivary flow was significantly increased in patients receiving the SOC intervention following extubation. It is worth noting that participants and caregivers credited this average 8-day SOC intervention across ICU settings to the general ward as an important bridge to resuming total oral intake postextubation.

Two points warrant emphasis. First, 45.6% of participants (≥ 50 years) did not resume total oral intake by 21 days after extubation, calling for effective interventions [13, 23]. Resuming total oral intake is not spontaneous after extubation, taking 3 weeks or longer to recover, even for those with no preexisting neuromuscular disease or swallowing dysfunction. A follow-up at 3 weeks postextubation is indicated to identify patients who may benefit from referrals (e.g., rehabilitation or ear-nose-throat services) to enhance the return of oral intake function. Moreover, as a secondary prevention, the SOC intervention helps ICU patients following endotracheal extubation to resume total oral intake, especially for those ≥ 65 years old. Future randomized controlled studies should verify our findings, understand the factors that may magnify or attenuate the SOC intervention effects, and define the SOC intervention effects in various ICU populations.

Second, targeting oral lubrication and oral sensation appears beneficial as reduced salivary flow was common among patients following endotracheal extubation, and 37.4% of our participants met the criteria for hyposalivation 2 days postextubation. This finding is consistent with a report that salivary flow was nearly absent in 24 intubated patients during their ICU stay [24]. Hyposalivation may be caused by lack of oral intake, systematic volume disturbances, medication, and stress with underlying disease. Saliva is also not distributed through the oral cavity in a supine position [25]. Indeed, oral lubrication (i.e., endogenous saliva or exogenously administered oral care products or food particles lubricate tooth-tooth, tongue-palate, and tongue-mucosa interfaces) plays a vital role in effective functioning of swallowing, chewing, and tactile perception [26].

Moreover, oral sensation is important as the oral cavity has a rich somatosensory innervation and stimulating these sensory receptors in the tongue and parts of the oral cavity may improve proprioception and oral sensorimotor control in swallowing [27]. Our SOC intervention targeted oral lubrication and oral sensation, providing both salivary gland massage and toothbrushing to produce pressure and vibratory mechanical stimulation of sensory receptors in the tongue, periodontal ligaments (through pressure on the teeth), gingiva, and palate, modulating salivary flow rate [17] and somatosensory function [15, 28, 29], all of which may improve oropharyngeal transit (i.e., swallowing efficiency). This pathophysiological mechanism is preliminarily supported by our positive findings on resuming total oral intake and increasing salivary flow. Further studies are needed to integrate the instrumental evaluation of swallowing to clarify the mechanism of how each SOC component works.

### Limitations

Although our study tested the first intervention to successfully help ICU patients resume total oral intake following endotracheal extubation, it had important limitations. First, without instrumental evaluation of swallowing, it is difficult to determine the physiology underlying impairments, to what extent participants differed individually and by group, and how the SOC intervention changed swallow physiology and airway invasion to increase the odds of resuming total oral intake. Second, we did not randomize participants to the study groups, which had some substantial differences at baseline. Although these differences were carefully controlled in the Cox proportional hazard model, the control group might have been favored by the SOC participants being older and having a worse baseline intake status, minimizing the intervention effect. Third, the SOC was designed as a multicomponent “bundled” intervention, making it difficult to determine which component was most effective. Fourth, participants were recruited from one medical center with exclusion criteria, and only 66.3% of participants (128/193) completed all salivary flow tests (22 were reintubated, 10 died, 21 were discharged, and 12 dropped out). These factors might have limited the generalizability of our findings.

## Conclusions

Despite many recent advances in ICU practice, management of dysphagia after extubation remains a significant challenge to healthcare providers and carries a significant weight of morbidity and mortality. Our study results show that a daily 15.4-min, nurse-administered SOC intervention comprising toothbrushing/salivary gland massage, oral motor exercise, and safe-swallowing education significantly increased the odds of resuming total oral intake for patients who had been successfully extubated after prolonged endotracheal intubation and had no preexisting neuromuscular disease or swallowing dysfunction. Age matters with the SOC intervention, which was more effective for those at least 65 years old.

## Supplementary information

**Additional file 1.** Swallowing and Oral Care (SOC) Intervention Protocols

## Data Availability

The datasets used and/or analyzed during the present study are available from the corresponding author on reasonable request.

## Abbreviations

FOIS: Functional oral intake scale
ROM: Range of motion
SOC: Swallowing and oral care
