# Supplementary material for: Effects of a swallowing and oral care intervention for patients following endotracheal extubation: a pre- and post-intervention study
Source: Crit Care. 2019 Nov 9;23:350. doi: 10.1186/s13054-019-2623-2 (PMC6842457; doi:10.1186/s13054-019-2623-2)
Supplement: Supplementary file 1 — Additional file 1. Swallowing and Oral Care (SOC) Intervention Protocols [file 13054_2019_2623_MOESM1_ESM.docx]

**Additional file**

| **Swallowing and Oral Care (SOC) Intervention Protocols** |
| --- |
| **Toothbrushing**   - SOC nurse rinses patient’s oral cavity with distilled water to soften sticky coated plaque; brushes the teeth/gum, tongue, and palate with a soft toothbrush; wipes the plaque off with a wet gauze; moistures patient’s lips with a thin layer of Vaseline^®^   **Salivary gland massage**   - SOC nurse gently massages patient’s cheek area in front of ears, moves along the chin under jaw and tongue areas, and gently presses 5-10 times in the submental area with both fingers to “milk” the parotid gland, the submandibular gland, and the sublingual gland |
| **Oral motor exercise of lips, tongue, jaw, and cheeks**   1. Active-assistive range of motion (ROM)  - Ask patient to purse the lips and spread them to the both sides of the face; move tongue forward/right/left out of the mouth, and retract back to the mouth; open the mouth widely; inflate and deflate the cheeks; pronounce /sh/- /sh/- /sh/ and extend the last /sh/ as long as possible. Each with 3, 5, or 10 repetitions, depending on participants’ tolerance  1. Resistive ROM when tolerated  - Ask patient to push lips outward against the force of the tongue depressor; push tongue to right and left cheeks and against the SOC nurse’s pressure on their cheeks; open the mouth to the maximum degree against the nurse’s pressure; inflate cheeks against nurse’s pressure; blow out the party horn. Each with 3, 5, or 10 repetitions, depending on participants’ tolerance |
| **Safe-swallowing education**   - Explain the signs and symptoms of unsafe swallowing - Advise on safe-swallowing strategies, including sitting up during oral intake, no feeding in a drowsy or sleepy state, strategies on modifying dietary texture and viscosity |
